# Water-Processable, Stretchable, and Ion-Conducting Coacervate Fibers from Keratin Associations with Polyelectrolytes

**DOI:** 10.1021/acssuschemeng.2c05411

**Published:** 2022-11-22

**Authors:** Jianwu Sun, Guillermo Monreal Santiago, Wen Zhou, Giuseppe Portale, Marleen Kamperman

**Affiliations:** † Polymer Science, Zernike Institute for Advanced Materials, University of Groningen, Groningen 9747 AG, The Netherlands; ‡ Polymer Science, Zernike Institute for Advanced Materials, University of Groningen, Groningen 9747 AG, The Netherlands; § Products and Processes for Biotechnology, Engineering and Technology Institute Groningen, University of Groningen, Groningen 9747 AG, The Netherlands; ∥ Macromolecular Chemistry and New Polymeric Material, Zernike Institute for Advanced Materials, University of Groningen, Groningen 9747 AG, The Netherlands

**Keywords:** keratin, complexation, fibers, processing, dry-spinning

## Abstract

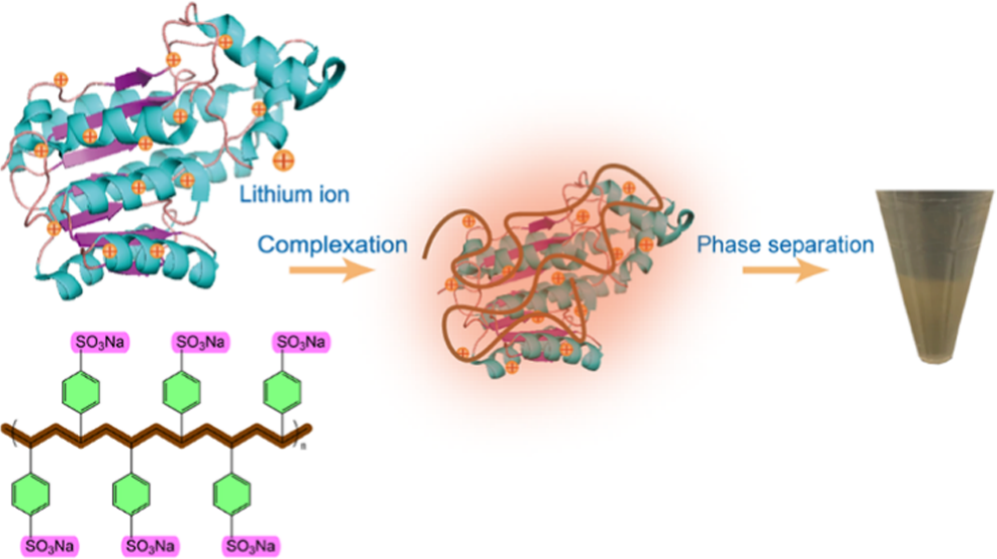

Keratin is one of the most abundant biopolymers, produced on a scale of millions of tons per year but often simply discarded as waste. Due to its abundance, biocompatibility, and excellent mechanical properties, there is an extremely high interest in developing protocols for the recycling of keratin and its conversion into protein-based materials. In this work, we describe a novel protocol for the conversion of keratin from wool into hybrid fibers. Our protocol uses a synthetic polyanion, which undergoes complex coacervation with keratin, leading to a viscous liquid phase that can be used directly as a dope for dry-spinning. The use of polyelectrolyte complexation allows us to use all of the extracted keratin, unlike previous works that were limited to the fraction with the highest molecular weight. The fibers prepared by this protocol show excellent mechanical properties, humidity responsiveness, and ion conductivity, which makes them promising candidates for applications as a strain sensor.

## Abstract 2

We report an efficient method to produce composite materials from keratin extracted from waste wool.

## Introduction

Protein-based materials have found a large number of uses throughout human history, from traditional textiles to current biomedicine. Their biodegradability, good mechanical properties, and natural abundance make them excellent candidates for many different applications.^1^ One of the most promising precursors for protein-based materials is keratin. Keratin is a structural protein found in all vertebrates, forming structures such as hairs, wool, hooves, and feathers. It has found applications in the manufacturing of advanced materials with very diverse applications, from tissue regeneration and drug delivery to food packaging and 3D printing.^2−4^ Keratin is produced in a scale of millions of tons per year as a byproduct of the animal industry,^5^ but due to a lack of efficient recovery methods, most of it ends up in landfills or as a pollutant, giving rise to environmental problems due to its slow degradation.^6^ Additionally, the extraction of keratin and the subsequent processing strategies are usually time- and energy-consuming processes that lead to materials with poor mechanical performance.^7^ For these reasons, there is a high interest to find environmentally friendly and cost-effective ways to improve the extraction and processing of keratin and to develop sustainable ways to make use of renewable keratin sources such as wool, feathers, and horns.

The association of keratin with polymers has been investigated for many years as a possible method for keratin extraction.^8^ It leads to the formation of composites that can be precisely tuned for different applications, combining some of the natural properties of keratin with new ones that come from the synthetic polymers. The associations between keratin and synthetic polymers are mainly classified into two types: blend formation (physical blending) and copolymer formation (chemical blending). Considering the poor miscibility of keratin with most synthetic polymers, direct physical blending is generally not possible due to weak inter- and intramolecular interactions.^9^ This can be circumvented by using compatible biopolymers, such as cellulose and chitosan, and dissolving them together with keratin in specific solvents, such as ionic liquids. This strategy has led to composites with enhanced mechanical properties, ascribed to intramolecular interactions formed during coprecipitations.^8^ In the realm of chemical blending, different functional groups and coupling agents have been introduced to further increase the compatibility and dispersibility of keratin. Shavandi et al. reported that graft polymerization of various monomers onto wools can greatly improve their physicochemical properties, allowing for a wide scope of products.^10^ Kaur et al. have reported the preparation of keratin bio-nanocomposite films with high tensile strength and elongation strain by chemical bonding during the modification of keratin in the presence of cellulose and montmorillonite nanoparticles.^11^ These efforts have improved the preparation and processability of materials based on keratin. However, they all still rely on time-, chemical-, and energy- consuming processes, which require precipitation, dialysis, chemical modification, and blending. Not many studies have focused on simplifying those steps to lower both their cost and environmental impacts and further facilitate the development of keratin-based materials.

Instead of using multiple postextraction steps, Cera et al. have recently demonstrated an easier processing strategy for keratin, which takes advantage of the presence of charges on its surface to prepare a viscous dope that can be spun into high-performance fibers.^12^ The positive charges, coming from the presence of Li^+^ ions absorbed onto the protein surface,^13,14^ can be neutralized by the addition of phosphate anions, leading to stronger protein–protein interactions and an increase in viscosity. Their methodology leads to keratin materials with interesting mechanical properties, but it only uses the larger and more crystalline peptides, discarding most of the extracted keratin. Similar strategies based on electrostatic interactions have also been applied to recombinant proteins to make advanced bioglues^15−17^ and fibers.^18^

Inspired by these examples of electrostatically driven phase separation as an intermediate step for the processing of protein materials,^19−25^ we decided to explore the direct extraction of keratin through the formation of a complex coacervate. Complex coacervates are polymer-dense phases formed through liquid–liquid phase separation, which is usually driven by the complexation of polyelectrolytes of opposite charges. The processing of coacervates is a greener methodology than that of conventional polymer melts since coacervates form spontaneously in water, can be processed at room temperature, and require only salt as a plasticizer.^19,20^

**Figure 1:**
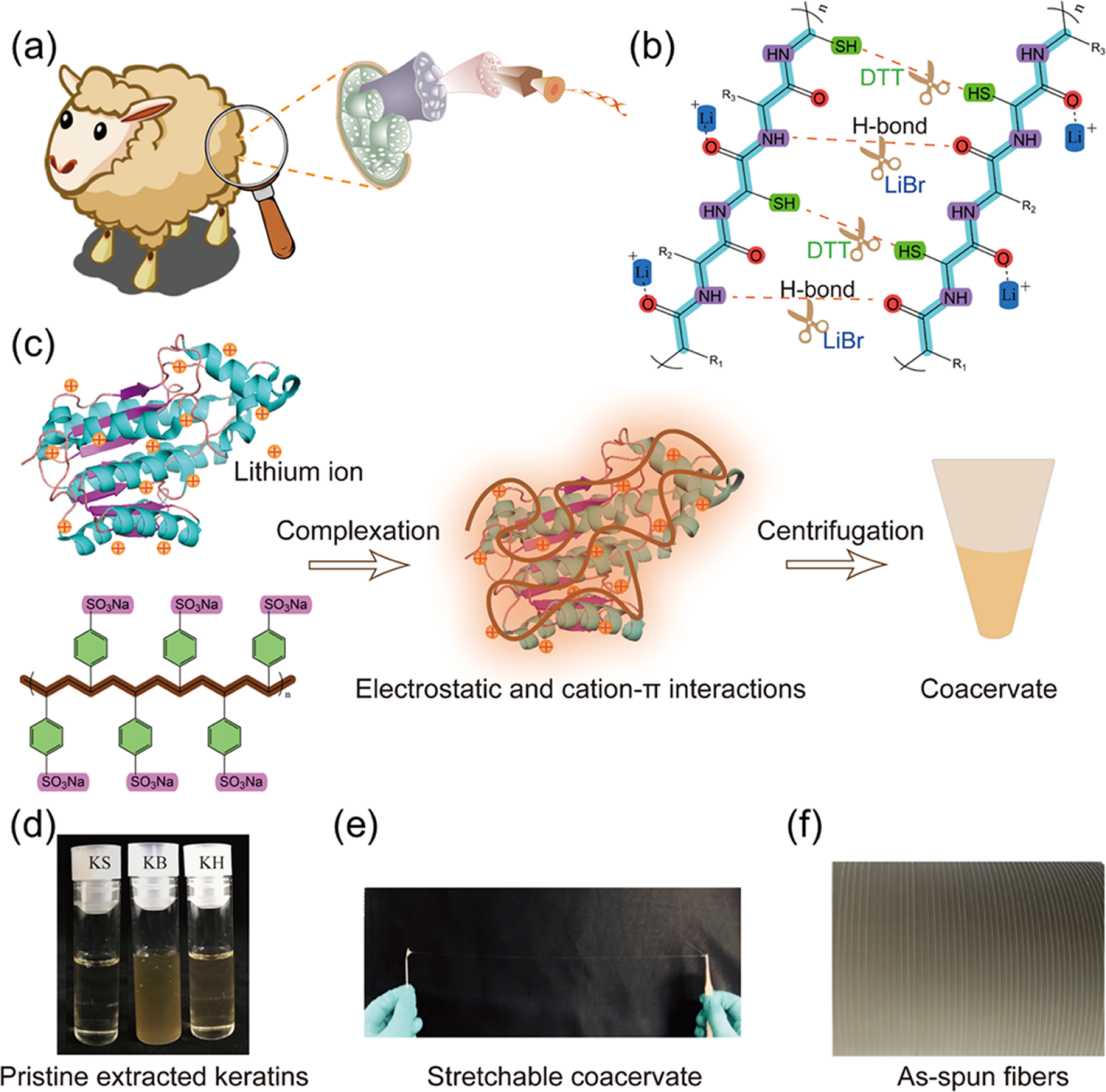
(a) Schematic diagram depicting the hierarchical structure of wool fibers. (b) Schematic representing the extraction mechanism of keratin: disulfide bond can be broken by the reducing agent DTT, while H-bond can be broken by LiBr salts. (c) Presentation of coacervate formation within positively charged keratin polypeptides and negatively charged PSSNa. (d–f) Photograph of the pristine extracted keratins: KS, KB, and KH; stretched coacervate fiber by hands; as-spun coacervate fiber by a syringe pump.

Here, we describe a facile and cost-effective method to complex pristine keratin with a synthetic polyanion to make coacervate fibers (Figure 1). First, we extract keratin from wool using a procedure known to lead to different phases containing positively charged keratin molecules. We characterize and study systematically these phases in terms of molecular weight, crystallinity, and thermal properties. Second, we use an aromatic polyanion to complex these keratins and form coacervates. By probing the effect of the LiBr concentration and polyanion content, we optimize the viscosity of the coacervates and dry-spin them into fibers. Lastly, we characterize the as-spun coacervate fibers, which exhibit good mechanical and ion-conducting properties. The keratin/polyanion coacervate developed in this study is a promising material that shows the potential of polyanion complexation as an efficient preparation method of keratin-based materials.

## Experimental Section

### Materials

Poly(sodium 4-styrenesulfonate) (PSSNa, *M*_w_ ∼ 200,000 Da, 30 wt % in H_2_O), lithium bromide (LiBr), dithiothreitol (DTT), and Laemmli buffer solution were obtained from Sigma-Aldrich and used without further purification. Composite wool consisting of 50% sheep wool and 50% alpaca wool was purchased from a local shop. The PSSNa solution was diluted to a concentration of 1 wt % using deionized water (reverse osmosis, conductivity < 10 μS/cm) before use.

### Extraction of Keratin from Wools

Keratin was extracted using a modified version of a previously described protocol:^12^ First, wools were cut into small pieces and then immersed in ethanol for 48 h before rinsing with deionized water. The wool fibers were air-dried, after which 10 g was transferred into a 250 mL flask with a 150 mL aqueous solution of LiBr (8 M) and DTT (0.1 M). The extraction was conducted at 90 °C for 36 h under a N_2_ atmosphere. Afterward, the keratin solution was obtained by fast hot filtration to remove insoluble residues. Finally, phase separation occurred when the keratin solution was stored at 4 °C for 12 h. We refer to the upper phase as a keratin supernatant (KS), while the lower phase was denominated a keratin bottom phase (KB). We use keratin homogeneous (KH) to refer to the keratin solution at room temperature before phase separation. To quantify the protein concentration in different phases, the dried solid keratin was obtained by dialysis (3.5 kDa cutoff) against water for 1 week before freeze-drying. The resulting protein concentrations in KH, KS, and KB were 1.6% (w/w), 1.1% (w/w), and 17.3% (w/w), respectively. This was confirmed using a Bradford protein assay. The total yield of keratin extraction was approximately 49% in weight.

### Preparation of Spinning Dope and Fabrication of Coacervate Fibers

The KS solution was mixed with 1 wt % PSSNa solution at different volume ratios and then centrifuged at 4500 rpm for 10 min at room temperature. The concentrated KB solution was diluted with a solution of LiBr (8 M, 0.1 M DTT) to 1 wt % before complexing with PSSNa. The corresponding coacervates at the bottom phase after centrifugation were labeled as KS/PSSNa and KB/PSSNa.

Coacervates prepared from a 1:1 keratin/PSSNa ratio were directly used as a spinning dope to fabricate fibers. The dry-spinning setup was custom-made and consists of a syringe pump (AL-4000, WPI) and a take-up roller. The diameter of the needle tip was 1.2 mm, and an extrusion speed of 0.2 mL/min was used. The rate of the roller was 9.4 mm/s. The diameter of the coacervate fibers collected in this way was 180–200 μm, as determined by optical microscopy.

### Characterization Techniques

#### Thermogravimetric Analysis (TGA)

Thermogravimetric analysis (TGA) was carried out on a TA instrument TGA Q50 by heating samples at 100 °C for 30 min to remove water and then rising to 700 °C in N_2_ at a rate of 10 °C/min. Differential scanning calorimetry (DSC) was performed on a TA instrument DSC Q100. The samples were all equilibrated at 100 °C for 30 min and then heated at 2 °C/min from −30 to 300 °C with a modulation amplitude of ±0.32 °C/min. For all DSC and TGA measurements, the samples were dialyzed beforehand to remove LiBr and then freeze-dried to remove water.

#### Attenuated Total Reflectance of Fourier-Transform Infrared (ATR-FTIR)

Attenuated total reflectance of Fourier-transform infrared spectroscopy (ATR-FTIR) measurements were performed with a PIKE MIRacle accessory with a diamond prism in a vertex 70 spectrometer (Bruker). The spectrum of 4500 to 400 cm^–1^ was acquired at a resolution of 4 cm^–1^ and 32 scans. Quantitative analysis of amide I (1600–1700 cm^–1^) was carried out using peak deconvolution methods using the software Origin (OriginLab Corporation). The three main peaks were assigned to the secondary structure of keratin: 1620 cm^–1^ (β-sheet), 1650 cm^–1^ (α-helix), and 1683 cm^–1^ (β-turn and random coil). A Gaussian model was selected for this band shape.

#### X-ray Diffraction (XRD)

The crystallinity index of pure keratin KS, KB, and KH was obtained with an X-ray diffractometer (XRD) (D8 Advance, Bruker, Germany) operated at a wavelength of 1.5418 Å, an operating voltage of 40 KV, and current flow of 40 mA. The freeze-dried samples were pressed into pellets before measurements. The test was performed between a 2θ value of 5 to −60° with 0.03° intervals at 1s per step. The crystallinity index (C.I.), which indicates the relative degree of crystallinity, was used to characterize the keratin materials:^26,27^ C.I. = (*I*_9_ – *I*_14_)/*I*_9_, where *I*_9_ is the maximal intensity with 2θ at around 9° and *I*_14_ is the minimal diffraction intensity with 2θ at around 14°.

#### Sodium Dodecyl Sulfate–Polyacrylamide Gel Electrophoresis (SDS-PAGE)

The molecular weights of the KS, KB, and KH solutions were analyzed using sodium dodecyl sulfate–polyacrylamide gel electrophoresis (SDS-PAGE).^28^ SDS-PAGE analysis was run on a 10% acrylamide gel and a Tris/Glycine/SDS buffer at a constant voltage of 100 V. Samples of the KS, KB, and KH solutions were prepared by mixing 10 μL of the diluted solution with 10 μL of Laemmli buffer solution containing 0.05 M DTT. The molecular weight of protein samples was estimated according to the molecular weight of standards (Tefco co., Ltd, Japan). All of the samples were tested at least three times.

#### Oscillatory Rheology

The rheological behavior of the coacervate was analyzed using a rheometer (AR 4000, TA Instruments) with a 20 mm parallel aluminum plate, and the gap was fixed at 500 μm. To determine the linear viscoelastic region, a strain sweep was performed from 0.1 to 10% at 1 Hz. Then, a frequency sweep was executed in the linear viscoelastic region (within 5% strain) to determine the elastic modulus (*G*′) and loss modulus (*G*″).

#### Scanning Electron Microscopy (SEM)

Scanning electron microscopy (FEI NovaNanoSEM 650) was used to characterize the morphology of keratin-related samples. The acceleration voltage was 10 KV, and the gold layer thickness was 20 nm.

#### Tensile Testing

Tensile testing was conducted with a universal testing machine (Instron, 3400 series) equipped with a 10 N load cell. All coacervate fibers KS/PSSNa and KB/PSSNa were stabilized for 24 h in a desiccator with different relative humidities (7% RH, 33% RH, 55% RH) before testing. The experiment was carried out at a gauge length of 15 mm under different constant extension rates (10 mm/min, 50 mm/min, 100 mm/min).

#### Wide-Angle X-ray Scattering (WAXS)

Wide-angle X-ray scattering (WAXS) experiments were carried out at the Multipurpose Instrument for Nanostructure Analysis (MINA) beamline at the University of Groningen. The diffractometer was equipped with a Cu rotating anode (λ = 1.5413 Å) using a sample-to-detector distance of 70 mm. Samples were prepared by gluing fibers on a stainless frame. The frame was positioned perpendicular to the beam and parallel to the equator axis of the detector. Data collection and analysis were conducted with Fit2D software.

#### Resistance Measurements

The resistance change of the fiber was measured through a Wheatstone bridge circuit. The voltage output (*V*_o_) of the Wheatstone bridge was measured by an electrometer, and the resistance of the fiber was calculated using the formula *R* = (*V*_s_ + 2*V*_o_)/(*V*_s_ – 2*V*_o_)*R*_0_, where *V*_s_ is the supply voltage (12 V) and *R*_0_ is the resistance of the Wheatstone bridge (*R*_1_ = *R*_2_ = *R*_3_ = 300 KΩ).

## Results and Discussion

### Keratin Extraction and Its Properties

We started our study by extracting pristine keratin from wools using a combination of LiBr and DTT.^12^ Previous studies have demonstrated that these two reagents can lead to the solubilization of the keratin present in wool. Li^+^ ions interact with carbonyl oxygens on the peptide chains to break hydrogen bonds between the polypeptide chains, while DTT is used to reduce the dense network of disulfide cross-links within keratin filaments (Figure 1b).^7,12−14^ Both of these reactions are reversible and do not affect the primary structure of keratin, so it is possible to use the proteins obtained in this way to form materials with a recovered hierarchical structure.^12^ After treatment with LiBr and DTT at a high temperature, we obtained a homogeneous pristine keratin solution (KH), which we cooled down after hot filtration, leading to phase separation between a supernatant (KS) and a bottom phase (KB) (shown in Figure 1d).

**Figure 2:**
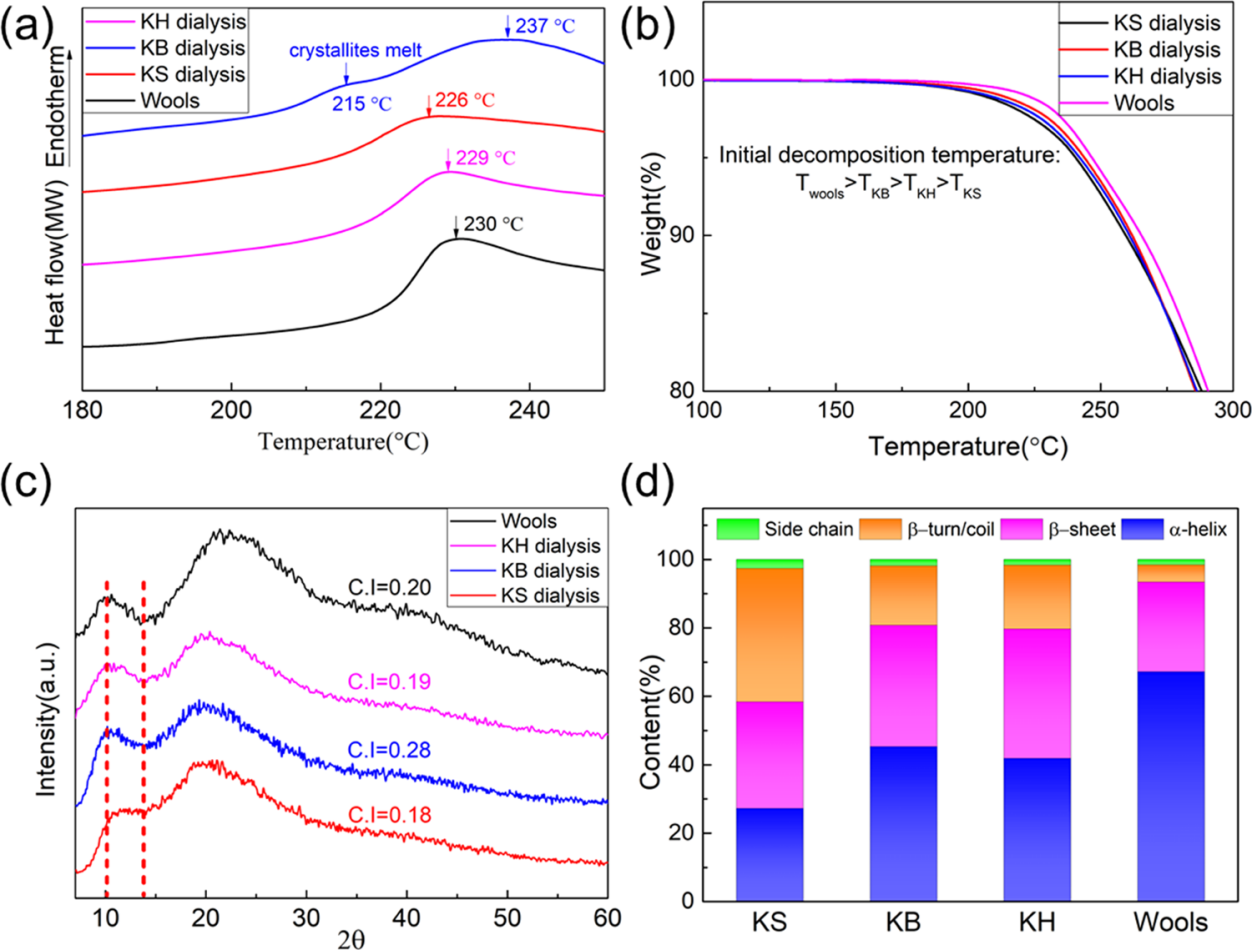
(a) DSC curves of KS, KB, KH, and natural wools. (b) TGA curves of KS, KB, KH, and natural wools. (c) XRD patterns of KS, KB, KH, and natural wools. (d) Quantitative analysis of secondary structures in KS, KB, KH, and wools.

To further understand the nature of this phase separation and later connect it to the properties of the final materials, we characterized the keratins present in each of the phases (KS, KB, KH). First, their molecular weight was determined by SDS-PAGE (Figure S1). We observed that KS contained only proteins with low molecular weights (25 KDa, 37–50 KDa), while larger proteins were also present in KB (25 KDa, 37–50 KDa, 50–75 KDa) and KH (25 KDa, 37–50 KDa, 50–75 KDa). We hypothesize that these larger peptides are more prone to self-assemble and restore part of their hierarchical structure, leading to their phase separation and the formation of KB. This is consistent with previous reports characterizing KB as a nematic phase, indicating fewer defects and a relatively high degree of crystallinity.^12^ To further characterize the different keratin phases, we studied them using thermal analysis. Figure 2a shows a DSC profile of KS, KB, KH, and natural wool, revealing the difference between them in terms of thermal properties. All of the samples exhibit a peak at around 230 °C, corresponding to both the denaturation of β-sheet crystallites and the decomposition of the polypeptide backbone. Another small peak at 215 °C, corresponding to the denaturation of α-crystallites,^24^ could only be observed in KB. This result corroborates that KB has a higher crystallinity than KS. In addition, the decomposition temperature of KB is increased to 237 °C as a result of its higher molecular weight and stronger intramolecular interactions. Surprisingly, we did not observe a peak at 215 °C for our sample of untreated wool. We hypothesize that in native wool, the α-helixes are organized into larger hierarchical structures, and their stability is increased until they degrade together with the peptide backbone. TGA results are shown in Figure 2b, showing that the initial decomposition temperature of KS, KB, KH, and natural wool follows the expected trend: *T*_wools_ (241 °C) > *T*_KB_ (236 °C) > *T*_KH_ (234 °C) > *T*_KS_ (232 °C).

To confirm that the differences in thermal properties were due to a different degree of crystallinity, we determined the crystallinity index (C.I.) of each of the keratin samples using XRD. A high C.I. value indicates high crystallinity of the samples.^26,27^ As shown in Figure 2c, KB has the highest crystallinity index (0.28), compared to KS (0.18), KH (0.19), or even natural wools (0.20). FTIR analysis of the amide I vibrations (shown in Figure S2 and summarized in Figure 2d and Table S1) confirmed that these differences in crystallinity were correlated to different secondary structures: KB had a higher content of organized and ordered conformations (α-helix + β-sheet, 80.8%) than KS (α-helix + β-sheet, 58.4%). The inverse was true for disordered structures (β turn/coil, 17.3% in KB and 39.0% in KS). In summary, these experiments confirmed that the keratin molecules of KB were more organized at the molecular level than those of KS.

**Figure 3:**
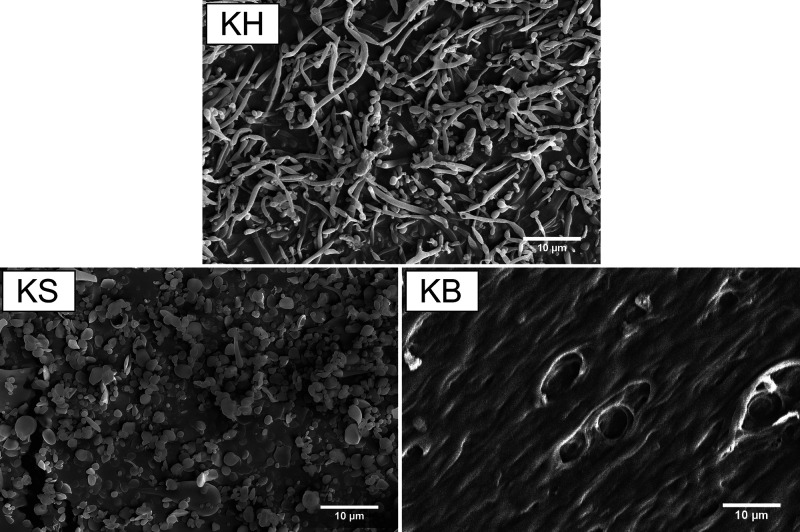
SEM images of KS, KB, and KH; scale bar is 10 μm.

To assess whether these differences at the molecular level resulted in a different hierarchical structure, we studied different keratin samples by SEM. KS, KB, and KH were dialyzed and freeze-dried to remove different salts and imaged by SEM. As shown in Figure 3, there were two sharply different components in KH before phase separation, one spherical and the other fibrous. After phase separation, the amorphous spheres were dominating over the filaments in KS, while the opposite was the case in KB. This shows that the crystallinity and ordered secondary structures present in KB are also translated into ordered structures at a micrometer scale, resulting in a more organized phase than KS.

#### Optimization and Characterization of Spinning Dope

**Figure 4:**
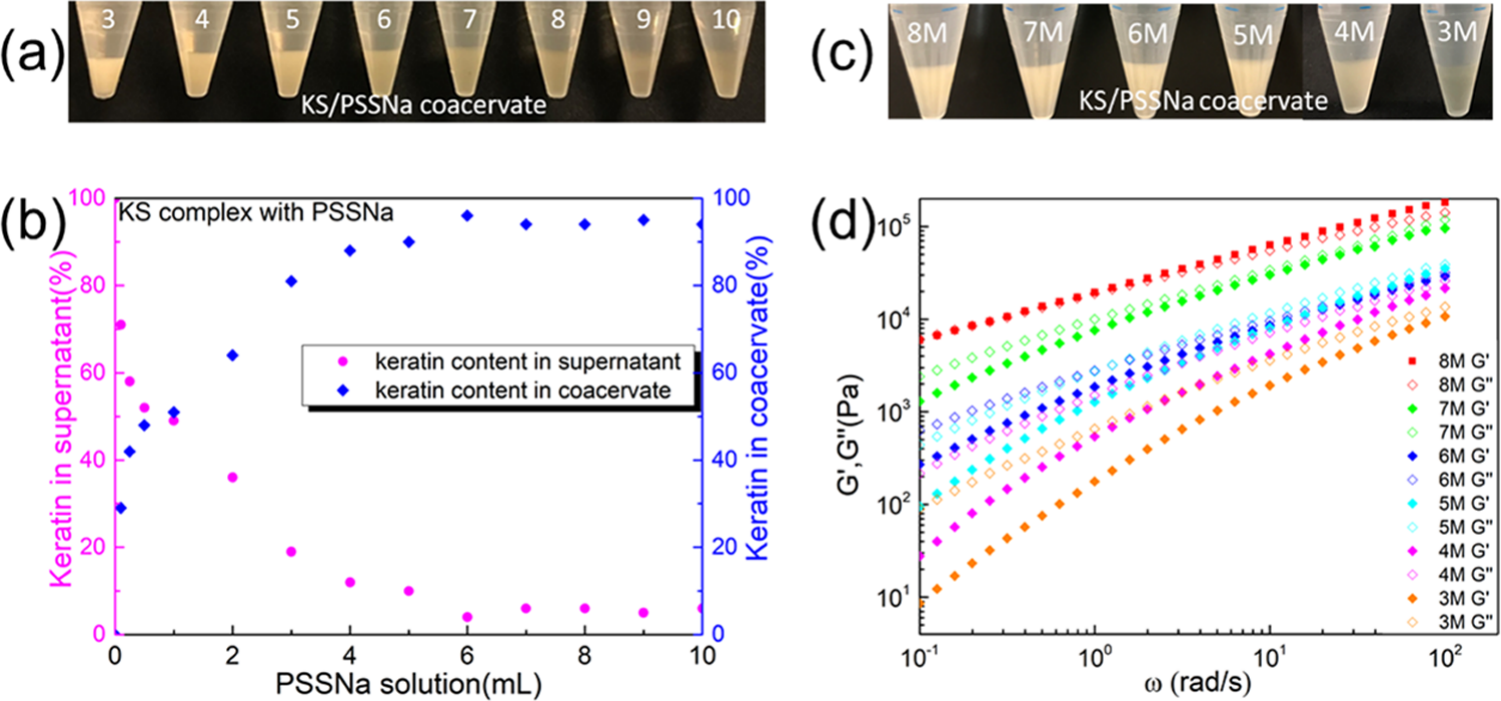
(a) Photograph of the KS/PSSNa coacervate, when 5 mL wt % KS 1 solution complexed with *x* mL 1 wt % PSSNa solution. (b) Protein percentage of supernatant and coacervate changes when adding more PSSNa solution into KS solution. (c) Photograph of the KS/PSSNa coacervate with different LiBr concentrations. (d) Viscoelastic properties of the KS/PSSNa coacervate with different LiBr concentrations.

After characterization of the different keratin phases, we set out to study their complexation with a polyanion to form a spinning dope that could be processed into fibers (Figure 1c,e). We selected for our study the polyanion poly(sodium 4-styrenesulfonate) (PSSNa) due to its ability to form coacervates^19,20^ and its structural similarity with sodium dodecyl benzene sulfonate (SDBS), a surfactant that was shown to interact with similar proteins through electrostatic and π–π interactions.^15,17^ Introducing the sulfonate groups as a polymer led to the formation of a coacervate (Figure 4a), a viscous liquid phase which could be centrifuged and decanted to separate it from the rest of the solution. This treatment, in contrast to most reported methods, allows us to directly form a spinning dope from the keratin in solution, instead of relying on time- and energy-consuming steps such as dialysis and freeze-drying. Remarkably, we could obtain coacervates not only from KB but also from the less crystalline KS (Figure 4a), therefore extracting all of the keratin from the system. This is in stark contrast to previously reported methods,^9^ where only the self-assembled phase (KB) was used, leading to a loss of 33% of the recovered keratin. The combination of electrostatic and π–π interactions and the multivalency of PSSNa was apparently critical to the process, as we did not observe any complexation with other common polyanions (alginate, cellulose, hyaluronic acid) and surfactants (SDS, SBDS).To further investigate this complexation and optimize the spinning dope for further applications, we first investigated the effect of different keratins: PSSNa ratios on coacervate formation. To ensure that all of the keratin molecules were available for complexation and to achieve a homogeneous coacervate phase, we first diluted KB to the same concentration as KS (1% w/w), and then, we titrated both solutions with increasing volumes of a 1% solution of PSSNa. As shown in Figures 4b and S3, the protein concentration in the supernatant decreased in both cases to less than 10% of its original value before the addition of PSSNa. Complete complexation was achieved in both cases with a volume ratio of 1:1 between PSSNa and keratin, and further additions of PSSNa barely changed the protein concentration in the supernatant beyond this point, indicating that almost all of the protein molecules had been extracted into the coacervate. Interestingly, the results from Figure 4b indicate that the ratio between keratin and PSSNa in the coacervate is different depending on the amount of PSSNa added (as more keratin seems to be extracted with the first additions of PSSNa than at larger volumes). This presents a simple strategy for tuning the keratin concentration in the final material. However, in this study we wanted to highlight that all keratin could be extracted from solution; therefore, we fixed the volume ratio between keratin and PSSNa to 1:1 from this point on.

Next, we investigated the influence of the LiBr concentration on the properties of the coacervate, a parameter known to affect the material properties of coacervates.^19,20^ In our case, the range of salt concentrations that we could access was limited by the charges of keratin. For keratin to interact with PSSNa, it needs to be positively charged, which in this case is due to the adsorption of Li^+^ ions onto its surface. To investigate this adsorption, we prepared different KS solutions at different LiBr concentrations. We observed that the keratin molecules aggregated and precipitated at salt concentrations of 3 M or lower (Figure S4 and Table S2). At higher concentrations, the keratin molecules remained in solution and did not interact with each other, stabilized by charge repulsion due to the adsorbed Li^+^.^12^ Therefore, we limited our study of different salt concentrations to values higher than 3 M.

The effect of salt in the coacervate can be clearly visualized as the coacervates become more transparent when lowering the LiBr concentration from 8 to 3 M (Figure 4c). This effect can be explained by the increased hydrophilicity of the coacervate caused by the weaker intermolecular interactions when lowering the charge density on the peptide chains. Changing the salt concentration also had an effect on the mechanical properties of the coacervates, as observed by oscillatory rheology (Figure 4d). The coacervates showed a stark decrease in both storage (*G*′) and loss (*G*″) moduli when lowering the salt concentration. Interestingly, this behavior is opposite from what is typically observed for polyelectrolyte complexes since in this case, LiBr is not shielding the interactions between polyelectrolytes but rather creates them by being adsorbed on the surface of keratin and creating positive charges. In this way, LiBr behaves more like a supramolecular cross-linker that enhances the complexation. This hypothesis is further supported by a liquid to solid transition (*G*′ > *G*″) that takes place at LiBr concentrations higher than 8 M, indicating that the electrostatic interactions are too large for the coacervate to flow. In general, this experiment shows the large range of viscoelastic properties that can be accessed by these keratin-based materials by only changing the salt concentration. In future applications, this modulation can be a great advantage since it allows for the fine-tuning of the mechanical properties of the spinning dope, adapting them to the desired processing conditions for each application.

#### Properties and Structural Analysis of the Keratin-Polymer Coacervate

**Figure 5:**
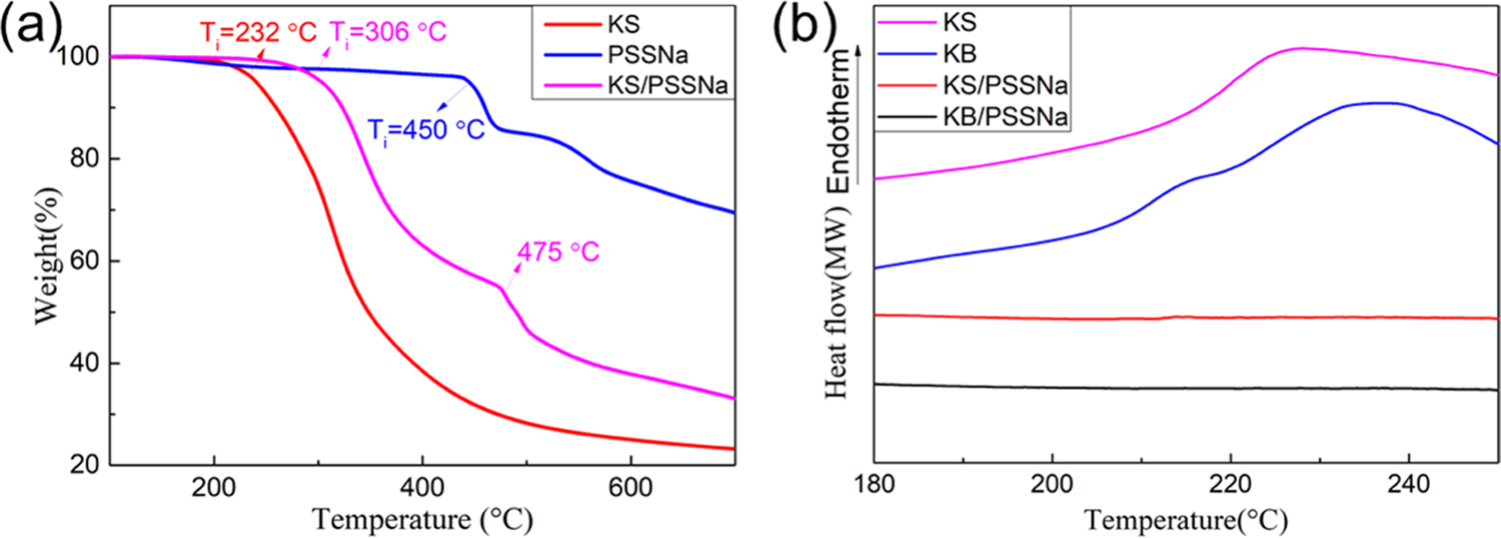
(a) TGA curves of KS, PSSNa, and KS/PSSNa. (b) DSC curves of KS, KB, KS/PSSNa, and KB/PSSNa.

To gain more insights into the interactions between polyelectrolyte and keratin, we studied the KS/PSSNa and KB/PSSNa coacervates after dialysis and freeze-drying by DSC and TGA. As shown in Figure 5a, the degradation temperature of both polypeptide chains and PSSNa molecules was increased in the KS/PSSNa coacervate from 232 to 306 °C in the case of KS and from 450 to 475 °C for PSSNa. This evidence demonstrates that there are strong inter/intramolecular stabilizing interactions between polypeptide chains and PSSNa. For the KB/PSSNa coacervate, we did not observe anymore transition corresponding to α-crystallite denaturation that appeared in KB by itself, indicating that complexation had taken place at a molecular level (Figure 5b), disrupting the formation of crystalline domains. The thermal properties of both coacervates were similar although the degradation temperature of the KB/PSSNa coacervate (318 °C) was slightly higher than that of KS/PSSNa (306 °C), demonstrating that the keratin molecules from KB were still able to form stronger interactions than those from KS (Figure S5).

To further study the interactions between keratin and PSSNa, we used ATR-FTIR (Figure S6). We observed four distinctive absorption bands of the −SO_3_ moiety in the coacervates; however, these bands were significantly red-shifted compared to PSSNa alone, confirming intermolecular interactions with the charged keratin. Since PSSNa presents an absorbance at a similar frequency than the amide I absorption band, we could not use this technique to study the effect of coacervation in the secondary structure of the protein.

The morphologies of the KS/PSSNa and KB/PSSNa coacervates, as observed by SEM, are shown in Figure S7. In this case, we did not observe spheres or filaments but a homogenous structure, confirming a strong interaction between polypeptide chains and PSSNa at a molecular level. Finally, we performed elemental analysis in pellets of all of the samples containing keratin: KS, KB, KS/PSSNa, and KB/PSSNa (Figure S8). C, N, O, S, and Na elements are homogeneously distributed at the pellet surface, revealing that the PSSNa molecules were evenly dispersed through the coacervate. The relative atomic contents of the different elements are summarized in Table S3, where we can see an increase in O and S contents and a decrease in the N content in the coacervates due to the presence of PSSNa.

The combination of all of these results shows that the complexation between keratin molecules and PSSNa takes place on a molecular level and homogeneously through the coacervate, disrupting the ordered structure of keratin but endowing it with a higher thermal stability.

#### Mechanical Performance of the Coacervate Fiber and Potential Applications

**Figure 6:**
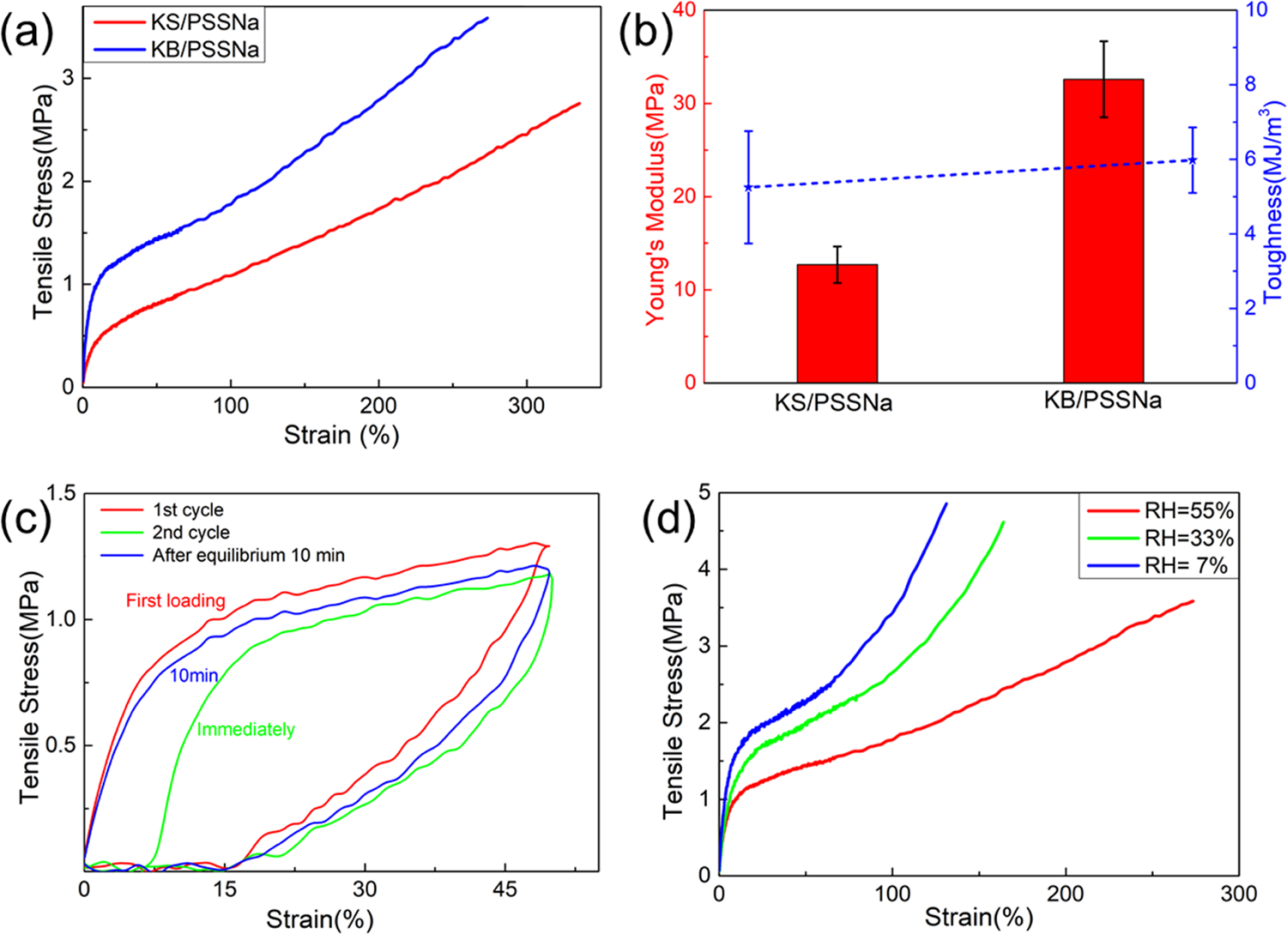
(a) Stress–strain plots of KS/PSSNa and KB/PSSNa fibers. Fibers were measured at 25 °C and 55 ± 5% RH. (b) Average Young’s modulus and toughness calculated from stress–strain curves of KS/PSSNa and KB/PSSNa fibers. (c) Stress–strain profiles of KB/PSSNa fibers subjected to cyclic testing at 50% strain: first cycle (red curve), second cycle (green curve, immediately after the first cycle), and third cycle (blue one, relaxed for 10 min after the second cycle). (d) Stress–strain curves of KB/PSSNa fibers under different relative humidities.

To demonstrate the potential of keratin-based coacervates for the production of materials, we used them as a dope for dry-spinning of fibers (Figure 1f). We were able to make fibers from these coacervates by simple extrusion, followed by drying, without any additional cross-linking steps. This method yielded fibers with elastomeric behavior (Figure 6a) and a good balance of strength and stretchability for both KS/PSSNa (2.8 ± 0.2 MPa, 340 ± 10%) and KB/PSSNa (3.5 ± 0.2 MPa, 270 ± 20%). In addition, these fibers presented a very high toughness (Figure 6b) (5 ± 2 MJ m^–3^ for KS/PSSNa, 6 ± 1 MJ m^–3^ for KB/PSSNa). The differences between the two different coacervates corroborate the results from the thermal tests: the crystallinity of KB/PSSNa is higher than that of its KS counterpart, which leads to a slightly higher strength and Young’s modulus and lower stretchability. However, the differences are remarkably small, highlighting the potential of this method to make materials from all of the extracted keratin and not just the more crystalline fraction. The fibers described here showed comparable mechanical properties to other keratin-based composites from the literature, with lower tensile strength and higher extensibility as a general trend (Table S4).

Protein-based materials typically exhibit mechanical properties that are sensitive to changes in strain rate and humidity, as well as hysteresis upon deformation and recovery. Interestingly, the KB/PSSNa fibers prepared using this protocol showed similar behaviors. First, they showed increased tensile strength and toughness and lower breaking strain at 100 mm/min than that at 10 mm/min (Figure S9), indicating that these materials become tougher upon fast impact and collision. Cyclic loading tests (Figure 6c) indicated that these fibers also showed hysteresis at a large deformation (50%). The initial cycle was mostly recovered over the course of 10 min, indicating that the noncovalent interactions responsible for the structure of the fibers were able to rearrange and dissipate energy efficiently. Finally, due to the high LiBr concentration, the KB/PSSNa fibers were able to absorb water from air, changing their mechanical properties. This humidity-dependent behavior can be seen in Figure 6d, where an increase in relative humidity from 7 to 55% led to a decrease in Young’s modulus, an increase in breaking strain, and a decrease in breaking stress (Table S5). The fibers were not completely resistant to water as submerging them in a water bath led to the dissolution of LiBr and the precipitation of keratin. For further uses where water resistance is required, we propose that the fibers could be treated with H_2_O_2_ to reform the disulfide cross-links between keratin molecules. This would have the additional advantage of allowing the fibers to be washed to recover the Li^+^ ions.

**Figure 7:**
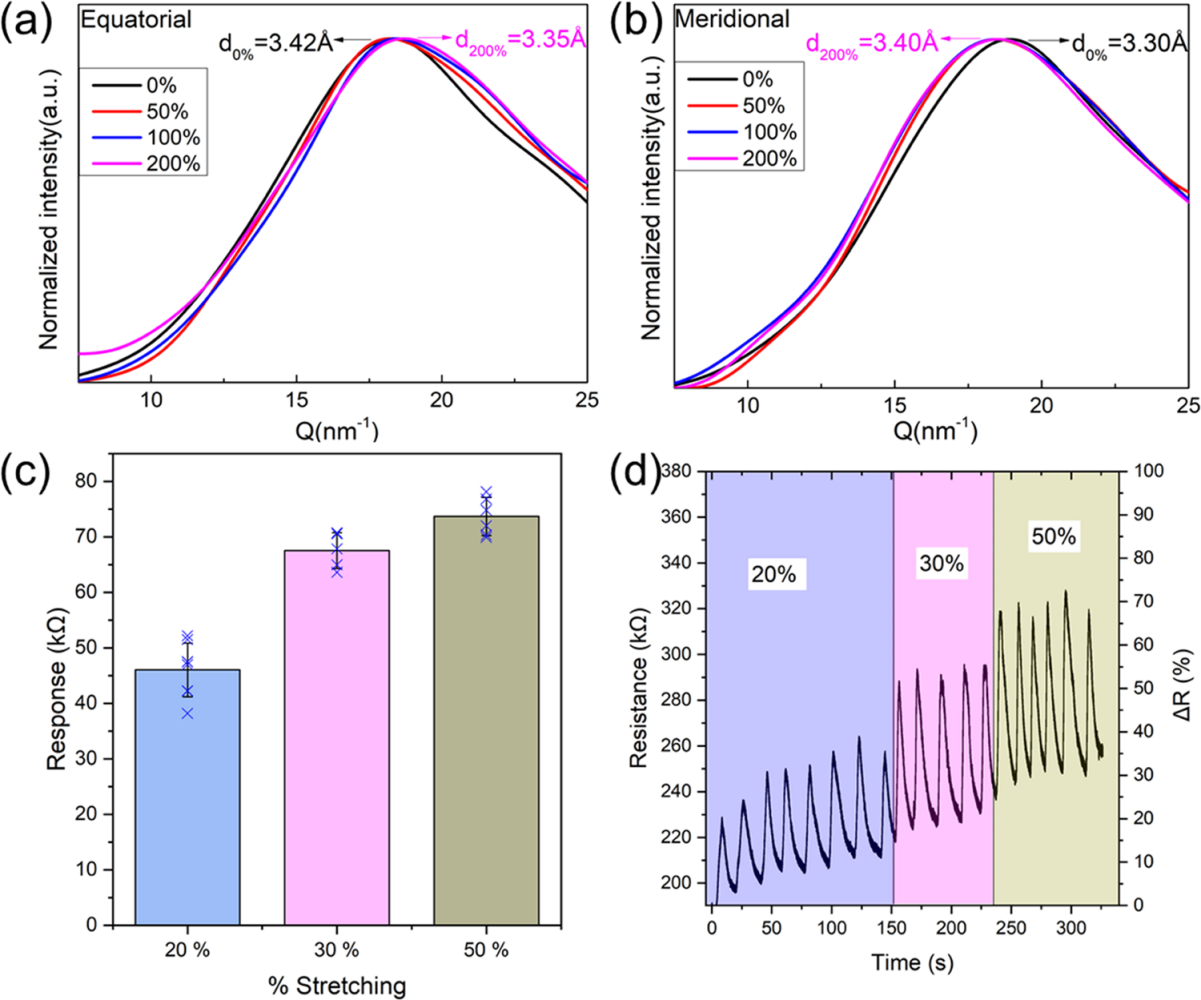
(a, b) One-dimensional scattering intensity profiles radially integrated at the equatorial direction and meridional direction. (c) Variation of resistance response (*R*–*R*_0_) of the KB/PSSNa fiber with different stretching ratios during the loading–unloading process. (d) Resistance change of the KB/PSSNa fiber with time during the loading–unloading process.

To probe the structural orientation in our coacervate fibers and study the effect of postspinning stretching on their structure, we used polarized optical microscopy (POM) and wide-angle X-ray scattering (WAXS). As shown in Figures S10 and S11, the KB/PSSNa fibers initially showed non-birefringent properties and a 2D WAXS pattern characterized by an isotropic halo, indicating the presence of a nonoriented amorphous internal structure. This indicates that the KB molecules, initially semicrystalline, lost their orientation and hierarchical self-assembly upon complexation with PSSNa (confirming the loss in crystallinity observed by DSC). In contrast to natural wool (Figures S12 and S13), the characteristic signals at 8.04 Å (interdistance between α-helix axes) and 3.91 Å (α-helix pitch) disappeared in the KB/PSSNa fibers. To quantitatively evaluate the development of the internal structure upon stretching, the two-dimensional data for KB/PSSNa fibers were integrated in the direction perpendicular to the fibers (equatorial) and parallel to the fibers (meridional). As shown in Figure 7a,b, the broad peak corresponding to the distance between nonbonded atoms slightly decreased from 3.42 to 3.35 Å in the equatorial direction, while it increased from 3.30 to 3.40 Å in the meridional direction, demonstrating the slightly extended polymer chains along the fiber axis.^29^ Since KB/PSSNa coacervates have an amorphous structure, the extended polymer chains induced by the drawing process could relax very fast to their original states, leading to a minor remnant shift in spacing even when the stretching ratio is up to 200%. This confirms that the orientation of the polymer chains in the fibers remained mostly isotropic and the crystalline structure of keratin was mostly lost during coacervation.

Since KB/PSSNa fibers contain some water and a large amount of LiBr, they are potentially useful as stress sensors due to its electrical conductivity (measured around 20,000 μS/cm). As a proof of concept of the sensing capacity of these materials, we observed that the electrical resistance of the fibers increased when they were stretched, and that the change in resistance was proportional to the amount of strain applied (Figure 7c,d). The initial resistance was mostly recovered when the stress on the fibers was removed, and although some hysteresis was observed over several cycles, we could perform more than 20 cycles at different deformations without losing the response. This property could be exploited for potential applications such as humidity sensors and wearable electronics.

## Conclusions

Inspired by the phenomenon of coacervation (liquid–liquid phase separation based on electrostatic interactions), we have developed an efficient method to complex positively charged keratin with a polyanion at a molecular level, generating a viscous coacervate that can be used to fabricate keratin/polymer composite materials. In this work, we have first extracted pristine keratin from wool, using LiBr and DTT. The extraction of keratin with these chemicals can either yield a homogeneous phase (KH) or two separate ones (KB and KS), depending on whether the keratins are left to self-assemble at low temperatures. We have characterized these three different phases, determining that KB contains keratins with a higher molecular weight and crystallinity and has stronger inter/intramolecular interactions than the other two phases, explaining why this phase has been used as a precursor for regenerated fibers before. Next, we have proven that both KB and KS can complex with a polyanion (PSSNa) to form homogeneous protein–polymer coacervates with improved thermal properties but lower crystallinity. The mechanical properties of these coacervates can be easily tuned by changing the concentration of LiBr to prepare solids or liquids with moduli that can vary in the range of 10^2^ Pa. Finally, we use these coacervates as precursors for the dry-spinning of fibers, which show excellent mechanical properties and responsiveness to humidity and strain rate. Furthermore, the prepared fibers are ion-conductive, making them a promising candidate for sensing applications.

The method developed here is remarkable for its simplicity as it only requires mixing and centrifugation to obtain a spinning dope from the pristine keratin, circumventing the limitations of previous solution- and solid-based processing methods. Furthermore, it allows for the use of all of the pristine keratins (both KB and KS), which results in an increased efficiency and lower waste compared to traditional methods.

## Supporting Information Available

SDS-PAGE pattern of protein standard and extracted keratin; FTIR spectra and peak deconvolution of amide I of KS, KB, KH, and natural wools; summary of secondary structures of amide I in KS, KB, KH, and wools; protein content upon titration of a KB (1 wt %) solution with PSSNa (1 wt %); photographs of KS solutions with LiBr concentrations from 8 to 1 M; TGA curves of KS/PSSNa and KB/PSSNa; FTIR spectrum of KS, KB, KS/PSSNa, and KB/PSSNa; SEM images of KS/PSSNa and KB/PSSNA; SEM-EDS images of KS, KB, KS/PSSNa, and KB/PSSNA; SEM-EDS analysis of keratin-related samples: elemental content comparison; stress–strain curves of KB/PSSNa fibers under different stretching rates; mechanical properties of KB/PSSNa fibers with different relative humidities; POM images of KB/PSSNa fibers; 2D WAXS pattern of the KB/PSSNa fiber; 2D WAXS pattern of the nature wool fiber; and 1D WAXS scattering profiles obtained from natural wools (PDF)
