# Supplementary material for: Water-Processable, Stretchable, and Ion-Conducting Coacervate Fibers from Keratin Associations with Polyelectrolytes
Source: ACS Sustain Chem Eng. 2022 Nov 22;10(48):15968–77. doi: 10.1021/acssuschemeng.2c05411 (PMC9727776; doi:10.1021/acssuschemeng.2c05411)
Supplement: Supplementary file 1 — sc2c05411_si_001.pdf [file sc2c05411_si_001.pdf]

Water-processable, stretchable, and ion-conducting coacervate fibers from keratin associations with polyelectrolytes

Jianwu Sun[1], Guillermo Monreal Santiago[2], Wen Zhou[3], Giuseppe Portale[4], Marleen Kamperman[1]**

[1] Polymer Science, Zernike Institute for Advanced Materials, University of Groningen, 9747 AG, The Netherlands.

[2] Polymer Science, Zernike Institute for Advanced Materials, University of Groningen, 9747 AG, The Netherlands. Current affiliation: Université de Strasbourg, CNRS, UMR7140, 4 Rue Blaise Pascal, 67081 Strasbourg, France.

[3] Products and Processes for Biotechnology, Engineering and Technology Institute Groningen, University of Groningen, 9747 AG, The Netherlands.

[4] Macromolecular Chemistry and New Polymeric Material, Zernike Institute for Advanced Materials, University of Groningen, 9747 AG, The Netherlands

Number of pages: 7

Number of figures: 13

Number of tables: 5

*Corresponding Authors:

Guillermo Monreal Santiago: monrealsantiago@unistra.fr

Marleen Kamperman: marleen.kamperman@rug.nl

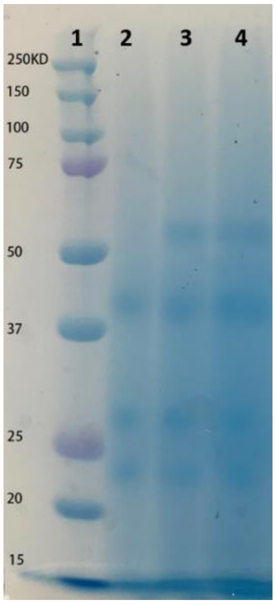

Figure S1. SDS-Page pattern of (1) protein standard, (2) KS, (3) KB,(4) KH

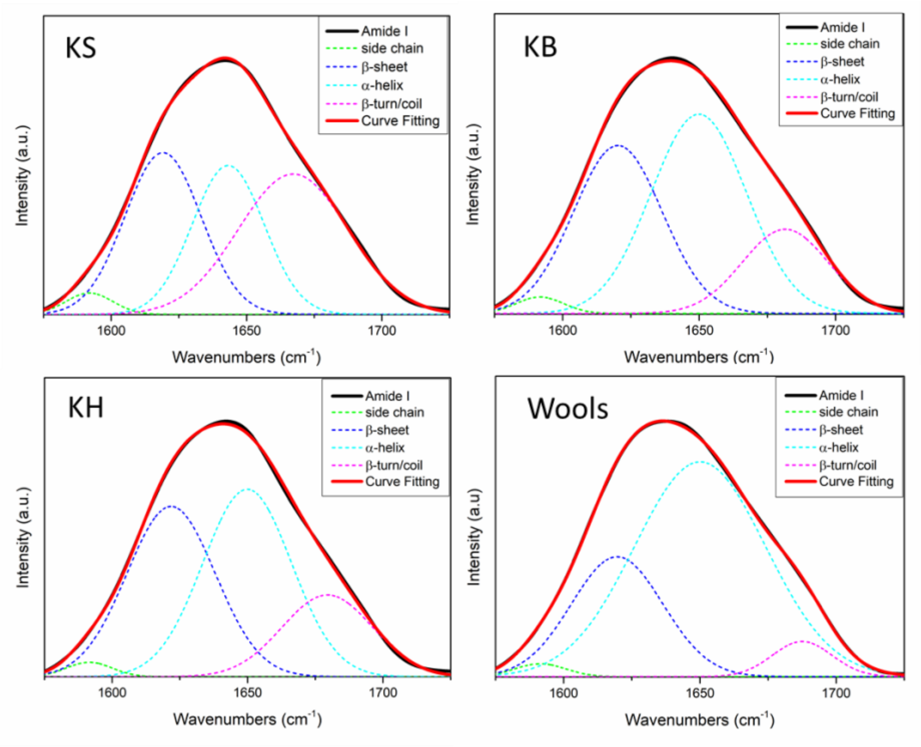

Figure S2. FTIR spectra and peak deconvolution of amide I of KS, KB, KH, Wools

Table S1. Secondary structures of amide I in KS, KB, KH, Wools were summarized as follows

Samples	Side chain	β-sheet	α-helix	β turn/coil
KS	2.6	31.2	27.2	39.0
KB	1.9	35.5	45.3	17.3
KH	1.7	37.8	41.9	18.6
Wools	1.6	26.2	67.2	5.0

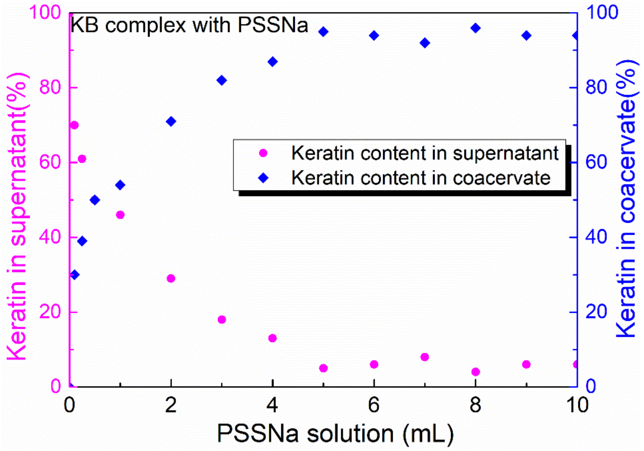

Figure S3. Protein percentage of supernatant and coacervate changes when adding more PSSNa solution (1 wt%) into KB (1 wt%) solution

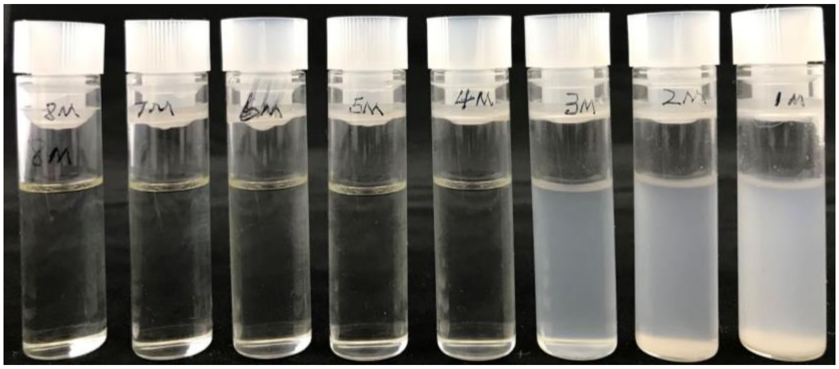

Figure S4. Photograph of KS solution with LiBr concentration from 8M to 1M

Table S2. the Z-average size of KS solution with different LiBr concentration

C_{LiBr}	8M	7M	6M	5M	4M	3M	2M	1M
Z-average(nm)	35	30	24	21	23	102	480	768
PDI	0.27	0.32	0.38	0.69	0.58	1.35	1.14	1.61
Transmittance(%)	100	100	100	100	100	99	74	54

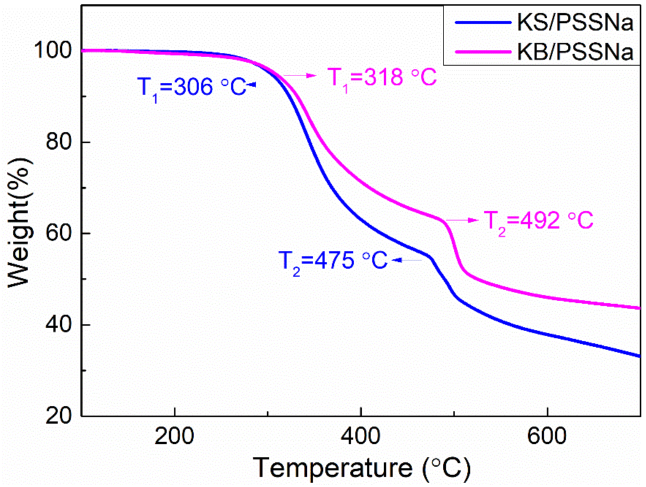

Figure S5. TGA curves of KS/PSSNa, KB/PSSNa

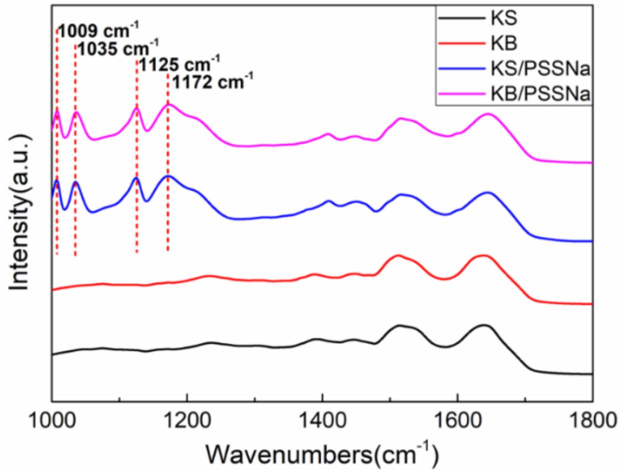

Figure S6. FTIR spectrum of KS, KB, KS/PSSNa, KB/PSSNa

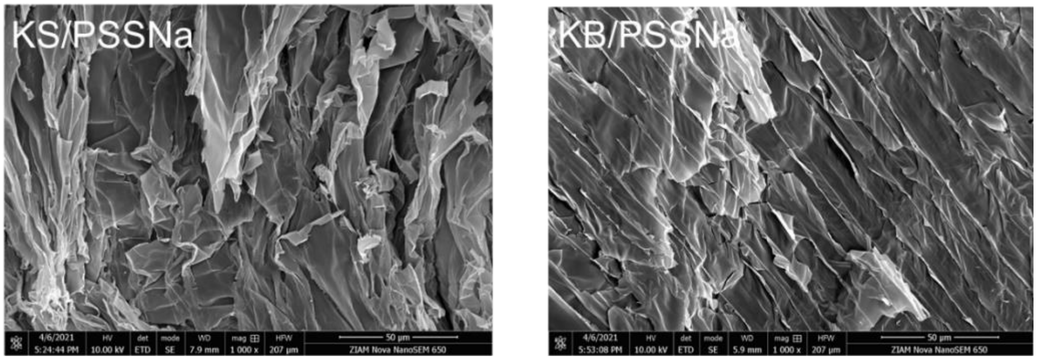

Figure S7. SEM images of KS/PSSNa, KB/PSSNA, scale bar is 50 µm

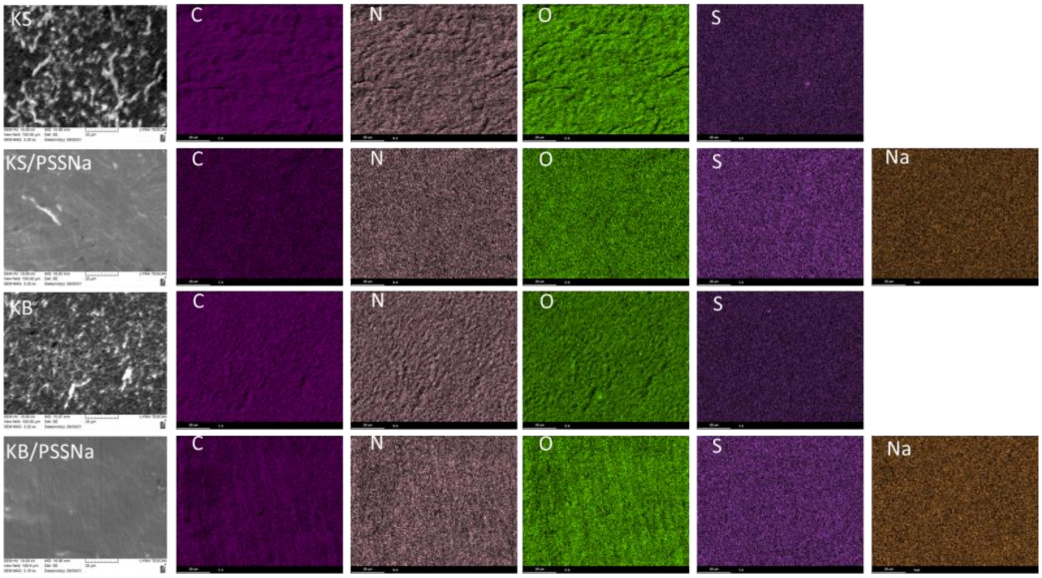

Figure S8. SEM-EDS images of KS, KB, KS/PSSNa, KB/PSSNA, scale bar is 20 µm

Table S3. SEM-EDS analysis of keratin-related samples: elemental content comparison

	C	N	O	S	Na
KS	61.1	20.1	14.5	4.3	0
KS/PSSNa	61.2	12.8	18.9	5.9	1.2
KB	63.2	19.5	14.0	3.3	0
KB/PSSNa	61.6	12.4	17.8	7.0	1.2

PSSNa: $(C_8H_7NaO_3S)_n$:C=61.5%; O=23.1% ;Na=7.7%; S=7.7%

Table S5. Mechanical properties of different keratin-based composite fibers

Additives	Keratin source	Keratin (wt%)	Tensile strength	Breaking strain
High-substituted hydroxypropyl cellulose [Supplementary Ref. 1]	Wools	80%	29 ± 6.2 MPa	80.96 ± 14.77 %
Montmorillonite nanoparticles [Supplementary Ref. 2]	Feathers	90%	6.0 ± 0.1 MPa	9.4 ± 0.5 %
Cellulose nanocrystals [Supplementary Ref. 2]	Feathers	90%	4.7 ± 0.1 MPa	21.1 ± 0.4%
Chitosan [Supplementary Ref. 3]	Wools	—	17.3 MPa	42.0%
PSS (this work)	Wools	50%	3.5 ± 0.2 MPa	270 ± 20 %

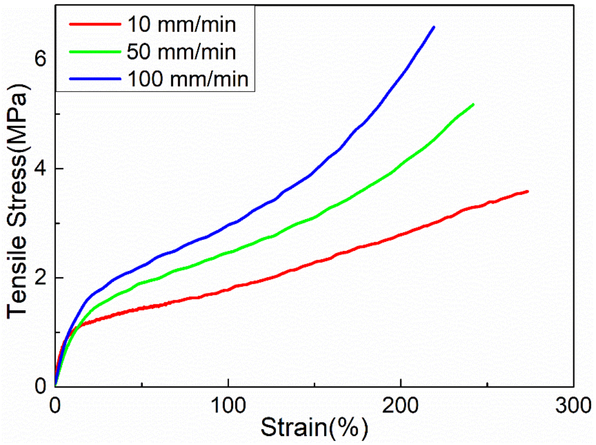

Figure S9. Stress-strain curves of KB/PSSNa fibers under different stretching rates

Table S5. Mechanical properties of KB/PSSNa fibers with different relative humidity

Relative humidity	Young's modulus (MPa)	breaking strain(%)	tensile strength(MPa)
55%	32.6±4.1	270.0±10.7	3.3±0.4
33%	36.3±3.6	160.0±8.6	4.2±0.6
7%	46.5±2.9	131.0±+4.8	4.9±0.2

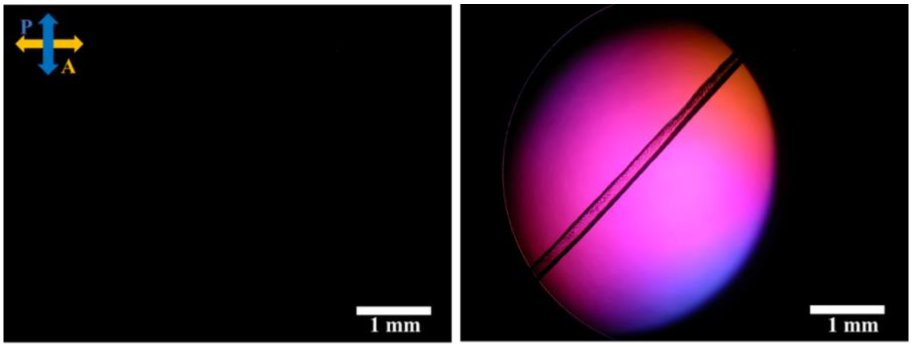

Figure S10. POM images of KB/PSSNa fibers: left without a tint plate; right with a tint plate (530nm)

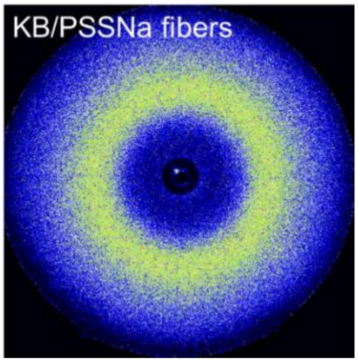

Figure S11. 2D WAXS pattern of the KB/PSSNa fiber

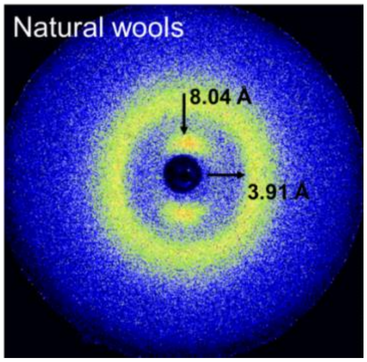

Figure S12. 2D WAXS pattern of the nature wool fiber, with black arrows pointing to the meridional and equatorial reflections

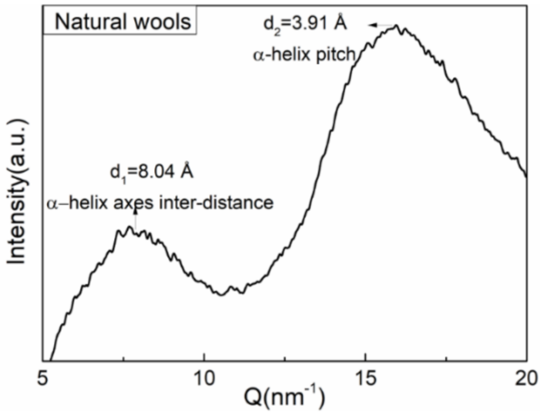

Figure S13. 1D WAXS scattering profiles obtained from natural wools, where 8.04 Å corresponds to α-helix axes inter-distance and 3.91 Å corresponds to α-helix pitch

Supplementary references:

1. Cao, G.; Rong, M. Z.; Zhang, M. Q., Continuous High-Content Keratin Fibers with Balanced Properties Derived from Wool Waste. *ACS Sustain. Chem. Eng.* **2020**, 8 (49), 18148-18156.

2. Kaur, M.; Arshad, M.; Ullah, A., In-Situ Nanoreinforced Green Bionanomaterials from Natural Keratin and Montmorillonite (MMT)/Cellulose Nanocrystals (CNC). *ACS Sustain. Chem. Eng*. **2018**, 6 (2), 1977-1987.

3. Ghosh, A.; Grosvenor, A.J.; Dyer, J.M. Improving the properties of chemically damaged wool fabrics with carbohydrate polymers. *J. Appl. Polym. Sci*. **2013**, 130, 3105–3111.
